# Fabrication, electrical characterization, and detection application of graphene-sheet-based electrical circuits

**DOI:** 10.1186/1556-276X-9-617

**Published:** 2014-11-15

**Authors:** Yitian Peng, Jianping Lei

**Affiliations:** 1 Jiangsu Key Laboratory of Design and Manufacture of Micro/Nano Bio-Medical Instrument, Southeast University, Nanjing 211189, China; 2 Suzhou Research Institute of Southeast University, Suzhou 215123, China

**Keywords:** Fabrication, Electrical characterization, Dielectrophoresis, Detection, Copper ions

## Abstract

The distribution of potential, electric field, and gradient of square of electric field was simulated via a finite element method for dielectrophoresis (DEP) assembly. Then reduced graphene oxide sheets (RGOS)- and graphene oxide sheets (GOS)-based electrical circuits were fabricated via DEP assembly. The mechanically exfoliated graphene sheets (MEGS)-based electrical circuit was also fabricated for comparison. The electrical transport properties of three types of graphene-based electrical circuits were measured. The MEGS-based electrical circuit possesses the best electrical conductivity, and the GOS-based electrical circuit has the poorest electrical conductivity among all three circuits. The three types of electrical circuits were applied for the detection of copper ions (Cu^2+^). The RGOS-based electrical circuit can detect the Cu^2+^ when the concentration of Cu^2+^ was as low as 10 nM in solution. The GOS-based electrical circuit can only detect Cu^2+^ after chemical reduction. The possible mechanism of electron transfer was proposed for the detection. The facile fabrication method and excellent performance imply the RGOS-based electrical circuit has great potential to be applied to metal ion sensors.

## Background

Graphene, a single layer of carbon atoms densely packed into a two-dimensional honeycomb structure, has received worldwide attention due to its extraordinary mechanical, electrical, thermal, and optical properties [1]. The attractive properties of graphene make it to be an ideal material for fundamental research and potential applications, such as electrical circuits [2], chemical and biological sensors [3], and composite materials [4].

Mechanical exfoliation of graphite on the pre-patterned electrodes is a popular method to fabricate high-quality graphene sheet-based electrical circuits. The extremely low throughput and lack of position precision severely limit the controllable fabrication of graphene sheet-based electrical circuits [5]. Reduction of graphene oxide provides an alternative method to produce large quantities of reduced graphene oxide sheets (RGOS) [6]. The ease of material processing, low cost of synthesis, and mechanical flexibility make RGOS become a perfect candidate for the fabrication of electrical circuits. In addition, RGOS exhibit a highly sensitive response to the outer environment. Thus, it is meaningful to explore the electrical properties of the RGOS for application. The RGOS was prepared by chemical reduction of graphene oxide sheets (GOS). Graphene sheets can be randomly deposited on the prefabricated electrodes by drop-coating suspension to fabricate graphene sheet-based electrical circuits. However, this method is not suitable for controllable fabrication of electrical circuits. Thus, directed assembly of graphene sheets at predetermined locations is required.

Dielectrophoresis (DEP) is a powerful technique for controllable fabrication of nanoelectronic devices, in which the DEP force is exerted on a polarizable object by non-uniform electric field [7]. DEP provides a simple, scalable, and low-cost method to position graphene sheets. Vijayaraghavan’s group use DEP for rapid assembly of individual flakes and nanoribbons of few-layer graphene into high-density electronic devices with a high yield [8]. Joung’s group explored high-yield fabrication of RGOS-based field effect transistors (FET). The RGOS in suspension were assembled between prefabricated gold source and drain electrodes via DEP [9]. Burg’s group carried out DEP deposition of few-layer graphene oxides between prefabricated electrodes [10].

Compared to the one-dimensional sensing materials including nanowire, nanotube, and nanoribbon, the two-dimensional graphene sheets used for detection have unique advantages because of their extremely large specific surface area, homogeneous functionalization, and high charge mobility and carrier concentration. As the surface is directly exposed to the ambient environment, the electrical property of graphene sheets is highly sensitive to the external disturbance. Sudibya’s group reported a FET sensor using protein-functionalized RGOS as the conducting and sensing channel [11]. Chen’s group reported an RGOS-based FET sensor, in which the RGOS was modified with gold nanoparticles [12]. The copper ions (Cu^2+^) are extremely harmful because of their toxicity that even a trace amount can pose detrimental damage to human health [13]. Thus, it is meaningful to find a facile, rapid, and sensitive method to detect the Cu^2+^.

In this paper, the RGOS, GOS, and mechanically exfoliated graphene sheets (MEGS)-based electrical circuits were fabricated and characterized, respectively. Then the three types of electrical circuits were applied for the detection of Cu^2+^. According to the relative change of electrical conductivity, the detection performance of graphene sheet-based electrical circuits for the Cu^2+^ in aqueous solution was evaluated. A possible mechanism for the detection was proposed.

## Methods

### Fabrication and electrical characterization

#### Fabrication of RGOS- and GOS-based electrical circuits

RGOS and GOS powder purchased from Nanjing XFNano Materials Technology Company (Nanjing, China) were used for the preparation of suspension. Five milligrams of RGOS powder was dispersed in 100 ml of N,N-dimethylformamide (DMF). The mixture was then ultrasonicated for 4 h. After nature sedimentation for 4 h at room temperature, the supernatant of solution was centrifuged at 1,000 rpm for 30 min to precipitate higher density aggregates. The supernatant in the centrifuge tubes was collected and used for the DEP assembly. The GOS were dispersed in de-ionized (DI) water via ultrasonication [14]. Five milligrams of GO powder was added into 100 ml of DI water. The mixture was then ultrasonicated for 2 h. The supernatant solution was then centrifuged at 1,000 rpm for 30 min. The supernatant in the centrifuge tubes was preserved for the DEP assembly.

**Figure 1:**
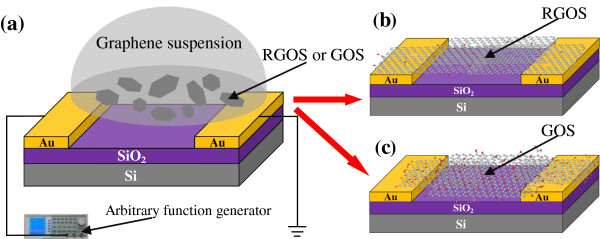
Schematics of (a) DEP assembly setup, (b) RGOS-based electrical circuit, and (c) GOS-based electrical circuit.

The array of micropatterned electrodes was fabricated via a lift-off process. The SiO_2_ layer of 300 nm thick was thermally grown on the N-doped Si substrate. A homogeneous layer of photoresist was then spin-coated on the SiO_2_ layer. Next, the photoresist layer was exposed using a pre-designed mask and developed, followed by thermal evaporation of Cr (5 nm) and Au (120 nm) layers. Then the substrate was immersed into the acetone to lift off the metal layer. The electrode array for DEP assembly was fabricated. The electrode array contains five pairs of electrodes numbered from 01 to 05. The gap size is 2 *μ*m.The DEP technique was used for the assembly of RGOS and GOS from suspension between the prefabricated Au electrodes. The DEP assembly was carried out on a probe station. Figure 1a shows the schematic of the DEP assembly setup.

**Figure 2:**
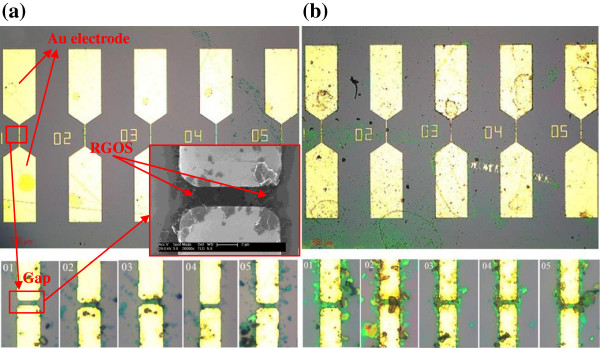
**Optical image of (a) RGOS and (b) GOS assembled between the Au electrodes (up).** Optical image zoomed gaps (down). The inset image shows a SEM image of RGOS assembled on the electrical circuit number 01.

The procedure of DEP assembly was described as follows: A drop of suspension (5 *μ*l) was dropped on the Au electrode pair. According to an arbitrary function generator, a sinusoidal wave signal was applied between the electrodes for a certain time and then removed. The strong electric field gradient assembles the graphene sheets aligned along the direction of the electric field gradient between the electrodes. The remaining suspension was rinsed immediately by ethanol and dried with nitrogen gas. The graphene sheet-based electrical circuit was fabricated when the graphene sheets bridge two Au electrodes. The adhesion between the graphene sheets and the Au electrodes was strong enough to fasten the graphene sheets in the same place after several cycles of washing and drying. Figure 1b,c shows the schematic of RGOS-based and GOS-based electrical circuits. During the DEP assembly of graphene sheets, the applied frequency, time, and suspension concentration were individually optimized to improve the assembly yield. The applied voltage amplitude has the strongest effect on the DEP force among all parameters [15]. For the DEP assembly of RGOS, the optimal voltage and frequency of sinusoidal wave signal were 3 V_p − p_ and 3 MHz. After applying to a pair of Au electrodes for 10 min, the signal was moved to the next pair. Then we obtained the RGOS-based electrical circuits. The GOS were also DEP assembled onto another substrate with electrode array. As the GOS were almost semiconducting, the applied voltage amplitude was improved to 20 V_p − p_. Figure 2a,b shows the assembly of RGOS and GOS between the Au electrodes after the RGOS- and GOS-based electrical circuits were fabricated.As shown in Figure 2, all of the Au electrodes were bridged with the RGOS or the GOS. As shown in the zoomed in image of Figure 2a, the assembled RGOS were mainly accumulated in the gap regions between the Au electrodes. Most of RGOS were distributed near the sharp corner of two Au electrodes, which revealing the sharp corner regions possess a higher electric field gradient. The inset image in the Figure 2a provides a SEM image of RGOS assembled on the electrical circuit number 01. As shown in the zoomed in image of a gap, the GOS that deposited across the gap regions showed a lot of overlapping layers and creases with clearly visible contours. Most of the GOS were assembled in the gap regions between Au electrodes, especially the sharp corner regions. These results denote that the corner regions have a high electric field gradient and then possessed the maximum DEP force.

**Figure 3:**
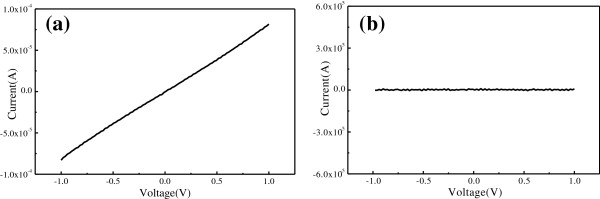
The representative I-V characteristics of (a) RGOS-based and (b) GOS-based electrical circuits.

Followed by the DEP assembly, the electrical transport of the RGOS- and GOS-based electrical circuits was measured from −1 to +1 V at room temperature. Figure 3a shows the representative I-V characteristic of RGOS-based electrical circuits. The characteristic is linear and symmetric [16]. The measured resistance is calculated about 12.3 *k*Ω. Figure 3b shows the representative I-V characteristic of the GOS-based electrical circuits. As the GOS are semiconducting, the I-V curve demonstrates the charge transport is limited.

#### Numerical simulation of DEP assembly of graphene sheets

The DEP force acting on the graphene sheets in a non-uniform electric field can be described by the following equation:

(1) $$\begin{array}{ll} {\vec{F}}_{DEP} & =\left(\vec{P}\cdot \nabla \right)\vec{E}=\left(VRe\left[\tilde{\alpha }\right]\vec{E}\cdot \nabla \right)\vec{E}\\ & =V\epsilon_{m}Re\left[K\left(\omega \right)\right]\left(\vec{E}\cdot \nabla \right)\vec{E} \end{array}$$

According to Equation 1, the time-averaged DEP force in an electric field cycle can be described as follows:

(2) $$\begin{array}{ll} \bar{{\vec{F}}_{DEP}} & =\frac{1}{4}V\epsilon_{m}Re\left[K\left(\omega \right)\right]\nabla \left[{\vec{E}}_{x}^{2}+{\vec{E}}_{y}^{2}\right]\\ & =\frac{1}{4}V\epsilon_{m}Re\left[K\left(\omega \right)\right]\nabla {\vec{E}}^{2} \end{array}$$

where $\vec{P}$ is the induced dipole moment, *V* is the volume of the graphene sheets, $Re\left[\tilde{\alpha }\right]$ is the real part of complex effective polarizability, $\vec{E}$ is the non-uniform electric field applied by the sinusoidal wave signal, and *Re*[*K*(*ω*)] is the real part of the Clausius-Mossotti (CM) factor, which is equal to

(3) $$K\left(\omega \right)=\frac{\epsilon_{p}^{*}-\epsilon_{m}^{*}}{\epsilon_{p}^{*}+2\epsilon_{m}^{*}}$$

where $\epsilon_{m}^{*}$ and $\epsilon_{p}^{*}$ are the complex permittivity of the medium and graphene sheets, respectively.

**Figure 4:**
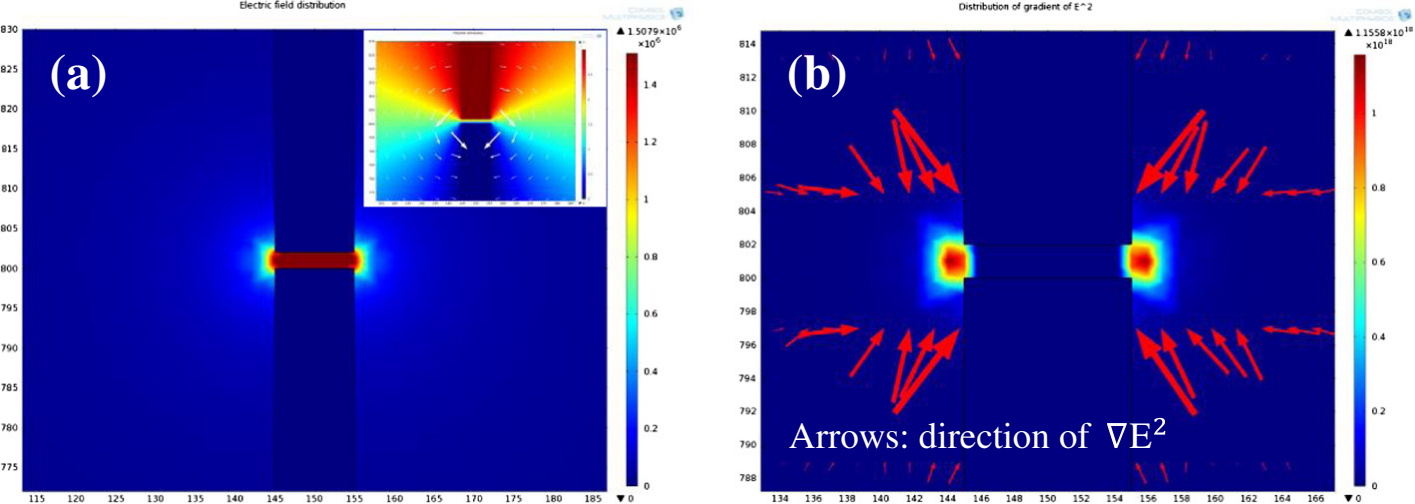
**Electric field distribution. (a)** Electric field distribution (inset image is the potential distribution and electric field lines). **(b)** The maps of ∇E^2^ distribution in the vicinity of Au electrodes.

In order to improve the yield and understand the mechanism of DEP, the effect of DEP force was investigated using FEM. The distribution of potential, electric field, and gradient of square of electric field (∇E^2^) was simulated [17]. The FEM model was built according to the geometrical parameters of Au electrodes. The potential difference of 3 V_p − p_ was applied between two Au electrodes. Electric field and ∇E^2^ distribution was shown in Figure 4.

The DEP force was exerted on the graphene sheets by the non-uniform electric field; thereby, the distribution of potential and electric field was simulated. As shown in the inset image of Figure 4a, the potential decreased gradually from anode to cathode along the electric field lines. The generated electric field strength reached the maximum value in the gap regions. The order of magnitude of electric field strength was as high as 10^6^ V/m between two Au electrodes. The electric field strength decreased sharply with the distance from electrodes, and the value was close to zero where the regions are far away from the Au electrodes. When the applied frequency was 3 MHz, the *Re*[*K*(*ω*)] was positive. Then the graphene sheets bear positive DEP force and were attracted to the regions of high electric field strength. This is the reason that the RGOS and GOS deposited preferably into the gap regions between two Au electrodes.

The DEP force exerted on the graphene sheets is directly proportional to ∇E^2^ according to Equation 2. Therefore, ∇E^2^ is an estimate of the DEP force in direction and magnitude [18]. Figure 4b shows the simulated maps of ∇E^2^ distribution in the vicinity of Au electrodes; the arrows indicate the direction of ∇E^2^. The deepest regions near the sharp corners of electrode pairs denoted the ∇E^2^ reached the maximum. Then DEP force also reached the maximum accordingly. Under the action of the DEP force, the graphene sheets were attracted to higher ∇E^2^ regions. Thereby most of graphene sheets were deposited near the sharp corner regions of Au electrodes that the experimental result has observed.

#### Fabrication of MEGS-based electrical circuit

**Figure 5:**
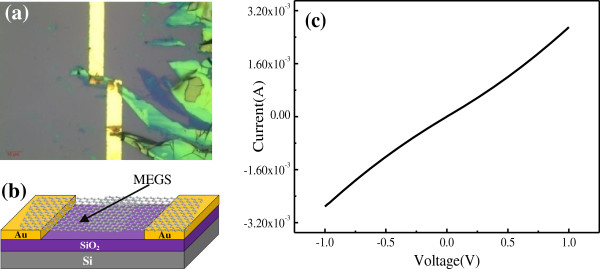
Optical image (a), (b) schematic, and (c) I-V characteristic of the MEGS-based electrical circuit.

As a comparison, the MEGS-based electrical circuit was also fabricated. The detailed procedure can be described as follows: A piece of fresh ‘Scotch’ tape was pressed firmly on a piece of highly oriented pyrolytic graphite (HOPG) for about 10 s. The tape was then gently peeled away with thick and shiny layers of graphite stuck to it. Next, this part of the tape with thick graphite flakes was refolded upon a clean adhesive section. The two layers of tape were pressed firmly together for several seconds and then gently unfolded so that the thickness of the graphite flake decreased nearly by half. After repeating the above exfoliation process for several times, a small amount of high-quality graphene sheets were obtained [19]. The SiO_2_ substrate with pre-patterned electrodes was ultrasonicated in the acetone, ethanol, and DI water for 10 min, and then dried with nitrogen gas. Next, the tape with the graphene sheets was pressed firmly on the prefabricated Au electrodes for about 30 min and then stripped gently. After repeating the above paste and stripping process of the tape for several times, the graphene sheets were attached randomly on the gap of Au electrodes. The MEGS-based electrical circuit was fabricated. Figure 5a,b shows the optical image and schematic of the MEGS-based electrical circuit.

Electrical transport of the MEGS-based electrical circuit was also measured from −1 to +1 V at room temperature. Figure 5c shows the I-V characteristic of the MEGS-based electrical circuit. The characteristic is not perfectly linear, which presumably arises due to contact barrier at the interface of the thin sheets and Au electrodes. The measured resistance was calculated about 370.4 Ω. The electrical conductivity of the MEGS-based electrical circuit was better than that of the RGOS-based electrical circuit. This can be attributed to surface and structural defects of the RGOS [20].

## Results and discussion

### Detection of Cu^2+^

In order to study the detection application of graphene sheet-based electrical circuits, the Cu^2+^ solutions were prepared by diluting copper sulfate pentahydrate into DI water. Then the Cu^2+^ solutions with different concentrations of 10 nM, 0.1 uM, 1 uM, 10 uM, 0.1 mM, and 1 mM were obtained by diluting the base solution. The detection performance of a graphene sheet-based electrical circuit is mainly characterized by its sensitivity and response time. The sensitivity *S* is defined as the relative conductance change in ambient air.

(4) $$S=\left(\left|G_{g}-G_{a}\right|\right)/G_{a}=\Delta G/G_{a}$$

where *G*_*a*_ is the conductance at the ambient condition, *G*_*g*_ is the conductance after the addition of solution, and Δ*G* represents the change of conductance after the addition of solution [21]. With respect to the response time, it is defined as the time taken for the relative conductance change to reach 90% of the next steady-state value from a steady-state value [22]. After detection, the remained solution on the graphene electrical circuit was thoroughly blown off with nitrogen gas. The current returned to the initial value after blowing which meant that the graphene sheet-based electrical circuit is able to be used for electrical detection repeatedly.

In order to characterize the detection performance of the graphene sheet-based electrical circuit for Cu^2+^ in aqueous solution, we measured its electrical conductance upon the addition of Cu^2+^ solutions with different concentrations. The applied voltage was fixed at a constant of 1 V. Thereby the measured current was equal to the conductance in value. The detection time was set to 250 s. The Cu^2+^ solution was dropped on the gap of two Au electrodes and the real-time responsive current of the graphene sheet-based electrical circuit was constantly monitored.

**Figure 6:**
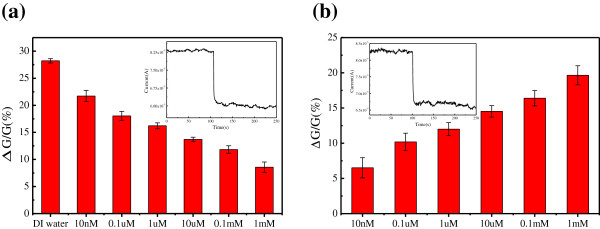
**Change of relative conductance of the RGOS-based electrical circuit against the concentration of (a) Cu**^**2+ **^**solutions, (b) Cu**^**2+**^**.** The inset images show the real-time current recordings of the RGOS-based electrical circuit with the addition of DI water and 10 nM Cu^2+^ solution, respectively.

As the Cu^2+^ diluted in DI water, the effect of DI water on the graphene sheet-based electrical circuit need to be considered. Therefore, the electrical detection for DI water was performed before detecting Cu^2+^ solutions. As shown in the inset image of Figure 6a, the current of the RGOS-based electrical circuit decreased instantaneously upon the addition of DI water and then reached a steady-state value within seconds. The inset image of Figure 6b shows the real-time current recordings of the RGOS-based electrical circuit with the addition of 10 nM Cu^2+^ solution. The currents also had similar changes with different levels for Cu^2+^ solutions of different concentrations. After the detection, the statistical results of relative conductance changes were provided. Figure 6a shows the relative conductance change of the RGOS-based electrical circuit against the concentration of Cu^2+^ solutions. The relative conductance change decreases with the concentration of Cu^2+^ solutions increasing from 10 nM to 1 mM.

The detection performance of the RGOS-based electrical circuit was stable. Whether dropping DI water or Cu^2+^ solutions with different concentrations, the current of the RGOS-based electrical circuit decreased immediately and then leveled off until it reached a steady-state value within seconds. Then the steplike current kept the constant value for a long recording time until the end of detection. As the response of the RGOS-based electrical circuit to the analytes was almost instantaneous, the response was super sensitive and the response time was just a few seconds.

The relative conductance change of the RGOS-based electrical circuit reached the maximum value when detecting DI water. As for Cu^2+^ solutions with different concentrations, the relative conductance change gradually decreases when the concentration of Cu^2+^ increases. The relative conductance change of the RGOS-based electrical circuit can be ascribed to the combined effect of DI water and Cu^2+^ on the RGOS when detecting Cu^2+^ solutions. The effect of Cu^2+^ on the RGOS-based electrical circuit can be considered to subtract the effect of DI water from Cu^2+^ solutions with different concentrations [23]. After removing the effect of DI water, the relative conductance increases gradually with the concentration of Cu^2+^ increasing from 10 nM to 1 mM, as shown in the Figure 6b. There was no doubt that the RGOS-based electrical circuit can be used to detect the Cu^2+^ of 10 nM.

**Figure 7:**
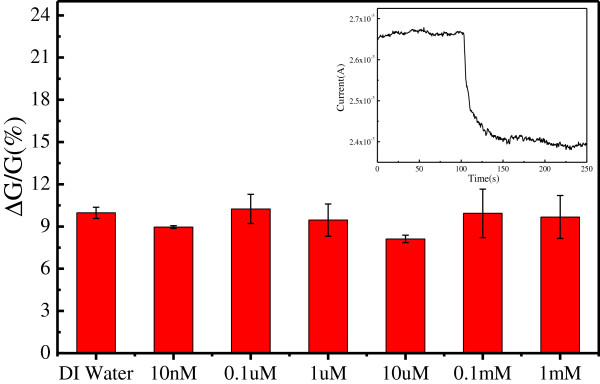
**Relative conductance change of the MEGS-based electrical circuit against the concentration of Cu**^**2+ **^**solutions.** The inset image shows the real-time current recording of the MEGS-based electrical circuit with the addition of DI water.

The detection performance of the MEGS-based electrical circuit was also demonstrated. Obviously, the analytes were still DI water and Cu^2+^ solutions with different concentrations. As shown in the inset image of Figure 7, the current of the MEGS-based electrical circuit also decreased immediately upon the addition of DI water. Different from the previous studies, the current did not reach a steady-state value within seconds but decreased continuously until the end of detection. When detecting Cu^2+^ solutions with different concentrations, the dynamic responsive current of the MEGS-based electrical circuit was also similar to that of detecting DI water. Figure 7 shows the statistical results of relative conductance change of the MEGS-based electrical circuit against the concentration of Cu^2+^ solutions. The relative conductance changes of the MEGS-based electrical circuit were approximately equal and random. It indicated that the MEGS-based electrical circuit was hard to detect with the Cu^2+^ in the aqueous solution.

**Figure 8:**
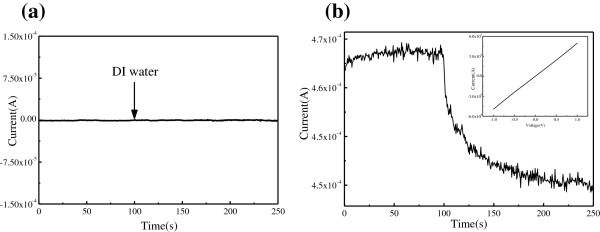
**Real-time current recording of the GOS-based electrical circuit with the addition of DI water. (a)** Before and **(b)** after chemical reduction.

Figure 8a shows the real-time current recording of the GOS-based electrical circuit with the addition of DI water. As the GOS were almost electrically insulting, the current is very small. The addition of DI water caused a subtle change of the current. The GOS was chemically reduced by hydrazine hydrate in a rapid heating oven, which was heated at 95°C for 24 h and then cooled to room temperature [24]. As shown in the inset image of Figure 8b, the I-V characteristic demonstrated the electrical conductivity of the GOS-based electrical circuit was improved after chemical reduction. The GOS-based electrical circuit was applied for the detection of Cu^2+^. Figure 8b shows the real-time current recording of the GOS-based electrical circuit with the addition of DI water after chemical reduction. When detecting Cu^2+^ solutions with different concentrations, the responsive currents were similar but the relative conductance changes were different. Thereby the GOS-based electrical circuit was able to detect the Cu^2+^ after chemical reduction.

**Figure 9:**
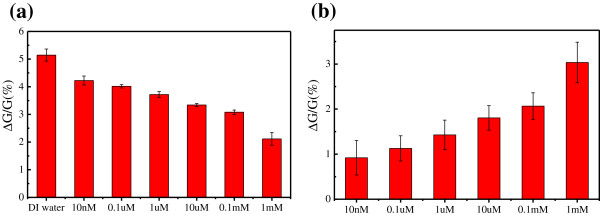
**Relative conductance change of the GOS-based electrical circuit.** Relative conductance change of the GOS-based electrical circuit against the concentration of **(a)** Cu^2+^ solutions and **(b)** Cu^2+^ after chemical reduction.

Figure 9a shows the relative conductance change of the GOS-based electrical circuit against the concentration of Cu^2+^ solutions after chemical reduction. In comparison with the detection of Cu^2+^ solutions with different concentrations, the relative conductance change of detecting DI water reached the maximum value. Besides, the relative conductance change decreased with the concentration of Cu^2+^ increasing from 10 nM to 1 mM. Considering the effect of Cu^2+^ on the GOS-based electrical circuit, the effect of DI water was subtracted. Figure 9b shows the relative conductance change of the GOS-based electrical circuit against the concentration of Cu^2+^ after chemical reduction. The relative conductance change gradually increased with the concentration of Cu^2+^ increasing from 10 nM to 1 mM. It was observed that the extent of the relative conductance change of the chemically reduced GOS-based electrical circuit was smaller than that of the RGOS-based electrical circuit when detecting the analytes. The main reason is that the RGOS were incompletely reduced after chemical reduction.

**Figure 10:**
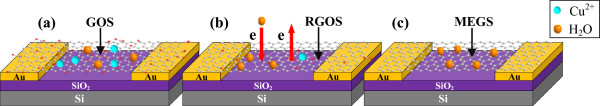
**Schematics of the (a) GOS-based (b) RGOS-based, and (c) MEGS-based electrical circuits when detecting Cu**^**2+ **^**solutions.**

Figure 10 shows the schematics of the GOS-based, RGOS-based, and MEGS-based electrical circuit when detecting Cu^2+^ solutions. It is well known that the structures of GOS, RGOS, and MEGS are different. The GOS contain hydroxyl, carbonyl, and carboxyl groups and are normally electrically insulating because of the abundant existence of saturated sp^3^ bonds, the high density of electronegative oxygen atoms bonded to carbon, and the distortion of graphitic network [25]. According to chemical reduction, the GOS can be reduced to the RGOS that have less functional group and restore the electrical conductivity, which allows the RGOS to work as the conducting channel of the electrical circuit. During the reduction process, carbon atoms get removed from the backbone of GOS, which generates the structural defects. In addition, line defects such as wrinkles and folds of the RGOS also create defects. The electrical properties of RGOS depend on the spatial distribution of functional groups and structural defects [26]. These residual functional groups which are not perfectly removed and structural defects are advantageous to absorb the Cu^2+^. On the contrary, the MEGS have a smooth and flawless surface and are difficult to be absorbed by the analytes [27]. The electrical conductivity of the MEGS-based electrical circuit was much more superior to that of the RGOS-based electrical circuit because a large amount of surface and structural defects exists in the RGOS. The RGOS-based electrical circuit has the best detection performance among all three electrical circuits.

Once the positively charged Cu^2+^ are absorbed on the surface of the graphene sheets, the electrical conductivity of graphene sheet-based electrical circuits was changed [28]. This can be explained by the charge transfer between the graphene sheets and Cu^2+^. The charged Cu^2+^ and water play the roles in acting as electron donors or acceptors which induce the change of charge carrier concentration in the graphene sheets [29]. The current of RGOS-based electrical circuit decreased immediately upon the addition of DI water. Since water molecules acted as an electron donor, the electrons transferred from the water molecules to the RGOS, which decreased the hole concentration in the RGOS and thereby decreased the current of RGOS-based electrical circuit [30]. When detecting Cu^2+^ solutions with different concentrations, the water molecules and the Cu^2+^ were simultaneously absorbed on the surface of the RGOS. In order to counteract the accumulation of positive charges from the Cu^2+^ ions, the electrons transferred from the RGOS to the Cu^2+^, which increased the hole concentration in the RGOS and thereby increased the current of the RGOS-based electrical circuit. Thereby, compared with the decrease of conductance when dropping DI water, the addition of Cu^2+^ solutions increased the conductance of the RGOS-based electrical circuit. The electrical conductivity of our RGOS-based electrical circuit increased with the concentration of Cu^2+^ increasing from 10 nM to 1 mM.

## Conclusions

The distribution of potential, electric field, and gradient of square of electric field of pre-patterned Au electrodes was simulated for DEP. The RGOS- and GOS-based electrical circuits were fabricated via DEP assembly. The MEGS-based electrical circuit was also fabricated for comparison. The three types of circuits have different electrical properties. The MEGS-based electrical circuit possesses the best electrical conductivity, and the GOS-based electrical circuit has the poorest electrical conductivity among the three circuits. For the detection the Cu^2+^, the RGOS-based electrical circuit can detect the Cu^2+^ at a concentration as low as 10 nM. The GOS-based electrical circuit can detect the Cu^2+^ only after chemical reduction. The water molecules and Cu^2+^ ions absorbed onto the surface of the RGOS play the roles of electron donators and acceptors for the detection. The facile fabrication method and excellent detection performance suggest that the RGOS-based electrical circuit has great potential to be developed into a metal ion sensor.

## Competing interests

The authors declare that they have no competing interests.

## Authors’ contributions

YT carried out the fabrication of the RGOS- and GOS-based electrical circuits via DEP assembly and the fabrication of the MEGS-based electrical circuit. JP carried out the electrical transport measurements and the detection application, the analysis of detection results and possible mechanism. Both authors read and approved the final manuscript.
